# RANK- and c-Met-mediated signal network promotes prostate cancer metastatic colonization

**DOI:** 10.1530/ERC-13-0548

**Published:** 2014-04

**Authors:** Gina Chia-Yi Chu, Haiyen E Zhau, Ruoxiang Wang, André Rogatko, Xu Feng, Majd Zayzafoon, Youhua Liu, Mary C Farach-Carson, Sungyong You, Jayoung Kim, Michael R Freeman, Leland W K Chung

**Affiliations:** 1 Uro-Oncology Research, Department of MedicineSamuel Oschin Comprehensive Cancer Center, Cedars-Sinai Medical Center8750 Beverly Blvd., Atrium 103, Los Angeles, California, 90048USA; 2 Department of SurgerySamuel Oschin Comprehensive Cancer Center, Cedars-Sinai Medical CenterLos Angeles, CaliforniaUSA; 3 Department of Biomedical SciencesSamuel Oschin Comprehensive Cancer Center, Cedars-Sinai Medical CenterLos Angeles, CaliforniaUSA; 4 Biostatistics and Bioinformatics Center, Samuel Oschin Comprehensive Cancer Center, Cedars-Sinai Medical CenterLos Angeles, CaliforniaUSA; 5 Department of PathologySchool of Medicine, University of AlabamaBirmingham, AlabamaUSA; 6 Department of PathologyUniversity of PittsburghPittsburgh, PennsylvaniaUSA; 7 Department of Biochemistry and Cell BiologyRice UniversityHouston, TexasUSA

**Keywords:** RANKL, RANK, c-Met, prostate cancer, metastasis, cancer dormancy

## Abstract

Prostate cancer (PCa) metastasis to bone is lethal and there is no adequate animal model for studying the mechanisms underlying the metastatic process. Here, we report that receptor activator of NF-κB ligand (RANKL) expressed by PCa cells consistently induced colonization or metastasis to bone in animal models. RANK-mediated signaling established a premetastatic niche through a feed-forward loop, involving the induction of RANKL and c-Met, but repression of androgen receptor (AR) expression and AR signaling pathways. Site-directed mutagenesis and transcription factor (TF) deletion/interference assays identified common TF complexes, c-Myc/Max, and AP4 as critical regulatory nodes. RANKL–RANK signaling activated a number of master regulator TFs that control the epithelial-to-mesenchymal transition (Twist1, Slug, Zeb1, and Zeb2), stem cell properties (Sox2, Myc, Oct3/4, and Nanog), neuroendocrine differentiation (Sox9, HIF1α, and FoxA2), and osteomimicry (c-Myc/Max, Sox2, Sox9, HIF1α, and Runx2). Abrogating RANK or its downstream c-Myc/Max or c-Met signaling network minimized or abolished skeletal metastasis in mice. RANKL-expressing LNCaP cells recruited and induced neighboring non metastatic LNCaP cells to express RANKL, c-Met/activated c-Met, while downregulating AR expression. These initially non-metastatic cells, once retrieved from the tumors, acquired the potential to colonize and grow in bone. These findings identify a novel mechanism of tumor growth in bone that involves tumor cell reprogramming via RANK–RANKL signaling, as well as a form of signal amplification that mediates recruitment and stable transformation of non-metastatic bystander dormant cells.

## Introduction

Bone is the most common site of prostate cancer (PCa) metastases. Metastatic lesions in bone are significantly associated with bone pain, hypercalcemia or hypocalcemia, pathological fracture, and spinal cord compression (Coleman 2001). Receptor activator of NF-κB ligand (RANKL)–RANK signaling has many crucial physiological roles in bone and other tissues (Hanada *et al*. 2009), and aberrant RANKL–RANK signaling in cancer and bone cells affects cancer bone colonization (Dougall 2011). We reported previously that β2-microglobulin (β2-M), a major histocompatibility protein co-receptor, promotes PCa cell osteomimicry by inducing RANKL and non-collagenous bone matrix proteins (Huang *et al*. 2006, Odero-Marah *et al*. 2008). β2-M-induced RANKL expression promoted epithelial-to-mesenchymal transition (EMT) in PCa cells (Zhau *et al*. 2008). Forced expression of β2-M in non-metastatic human breast, lung, and kidney cancer cells enhanced endogenous RANKL expression and induced EMT and bone and soft tissue homing (Josson *et al*. 2011). These findings support an important role for RANKL–RANK signaling in PCa metastasis.

Hepatocyte growth factor (HGF) and its receptor tyrosine kinase c-Met mediate cell motility, migration, increased tumor local invasion, and metastasis (Knudsen & Edlund 2004). Deregulation of the c-Met network is one of the most common mechanisms of solid tumor development (Birchmeier *et al*. 2003). The c-Met receptor cross talks with many membrane-localized signaling proteins, including EGFR, erbB2, erbB3, c-Src, and G-protein-coupled receptors, indicating the existence of numerous mechanisms of tumor cell escape from therapies directed at single oncogenic targets (Organ & Tsao 2011). c-Met inhibitors with differential selectivity have been developed based on their relative ATP competitive binding. Cabozantinib (XL-184), a recently FDA approved mixed c-Met, VEGFR2, KIT, and AXL inhibitor, proved highly effective against bone metastatic PCa (Smith *et al*. 2013). c-Met activation can be ligand-dependent or -independent and is associated with androgen receptor (AR)-deficient castration-resistant prostate cancer (CRPC; Verras *et al*. 2007) and AR-negative PCa stem cells (van Leenders *et al*. 2011).

Here, we describe a feed-forward loop, involving the induction of RANKL and c-Met signal and repression of AR, which drives RANK receptor-mediated bone metastasis. Novel RANK-mediated downstream master regulator (MR) transcription factors (TFs) were identified that coordinate PCa cell EMT, stemness, neuroendocrine, and osteomimicry phenotypes. We validated the roles of TFs and effector molecules in PCa metastasis with *in vivo* animal models guided by molecular imaging where abrogating RANK or its downstream c-Myc/Max or c-Met signaling network abolished skeletal metastasis in mice. Animal models also showed that RANKL-expressing PCa cells conferred bone colonizing and aggressive phenotypes to neighboring non-metastatic bystander cells by activating the RANK-mediated downstream signaling network. Significantly, RANKL and its downstream signaling network in primary human PCa tissues predict patient survival (Hu *et al*. 2013).

## Subjects and methods

### Cell culture

ARCaP_E_ and ARCaP_M_ cells established by our laboratory (Xu *et al*. 2006) represent stages of human PCa progression. They were maintained in T-medium (Invitrogen) supplemented with 5% FBS. LNCaP and LNCaP^Neo/RANKL^ were maintained in RPMI-1640 supplemented with 10% FBS. RAW264.7 cells, provided by Dr Neale Weitzmann at Emory University, were maintained in DMEM supplemented by 10% FBS.

### Cloning RANKL expression vectors

Human RANKL cDNA ORF (NM_003701) purchased from OriGene (Rockville, MD, USA) was amplified by PCR and subcloned into p3×FLAG-myc-CMV-25 (Sigma–Aldrich) at the NotI and XbaI restriction enzyme sites. The amplified human RANKL cDNA was subcloned into pCDH-CMV-MCS-EF1-copGFP (System Biosciences, Mountain View, CA, USA) plasmids at the XbaI and NotI restriction enzyme sites. The sequences of p3×FLAG-RANKL and pCDH-CMV-RANKL-EF1-copGFP were confirmed by DNA sequencing.

### Cell transfection, transduction, and gene deletion protocols

LNCaP cells were transfected with either p3×Flag-tagged RANKL or neo-control-plasmid (p3×FLAG-myc-CMV-25) cDNA (Sigma–Aldrich) for 48 h. The stable cell clones were selected in 400 μg/ml of G418 until individual colonies containing the transfected construct were confirmed by western blot analysis. The retrovirus packaging cells, 293GPG, were maintained in DMEM with 10% heat-inactivated FBS supplemented with tetracycline, puromycin, G418, and penicillin/streptomycin. The shRANK-1 and -4 retroviruses were generated using the retroviral small interfering RNA (siRNA) constructs pPower-hRK-1 (hRK-1) and pPower-hRK-4 (hRK-4) prepared using standard molecular cloning techniques and were used to infect ARCaP_M_ and LN^RANKL^ cells for 24 h in the presence of 8 μg/ml of polybrene. The oligosequences of RANK and control short hairpin RNA (shRNA) are as follows: shRANK-1, CCAGAAGATATGTGCTACCCA; shRANK-4, TGGGACGGTGCTGTAACAAA; and shCon, ACCATCTTAGTAGAGGTTGTT. The transfected cells as well as parental and tumor-derived LN^Neo-RFP^ cells were also transduced with firefly luciferase retroviruses (MSCV Luciferase PGK Hygro; Addgene, Cambridge, MA, USA) and selected with 200 μg/ml hygromycin. To evaluate the possible functions of key receptors and TFs in PCa metastasis, we conducted genetic deletion studies of c-Met and c-Myc and/or Max from LN^RANKL^ cells. c-Myc (sc-29226-V) and Max (sc-38080-V) shRNA viral particles were purchased from Santa Cruz Biotechnology, Inc. and c-Met shRNA viral particles (TRCN0000009851, TRCN0000040044, and TRCN0000121087) were purchased from Sigma–Aldrich.

### *In vivo* experiments

All animal procedures were performed according to an approved protocol from the Institutional Animal Care and Use Committee. LN^RANKL^, LN^Neo^, or LN^Neo-RFP^ cells (1×10^6^ cells/50 μl PBS) were tagged with the luciferase gene and inoculated intracardially or intratibially into 5- to 7-week-old male athymic nude mice (Charles River, Wilmington, MA, USA) as described previously (Odero-Marah *et al*. 2008). To study their interactions and recruitment, in some studies, cells were injected together or separately at different ratios or by a separate route. All mice were imaged weekly with either bioluminescence or fluorescence (red fluorescent protein (RFP): excitation, 570 nm; emission, 620 nm; a near-infrared MHI-148 organic dye: excitation, 783 nm; emission, 840 nm (Yang *et al*. 2010)) using Xenogen and scanned by X-ray by Luminar XR or X-ray microtomography (μCT) by Scanco vivaCT40 to examine the skeleton and determine the types of bone lesions.

### RT-PCR and quantitative real-time PCR

Total RNA from cells was isolated using an RNeasy Mini Kit (Qiagen) according to the manufacturer's instructions. cDNA was generated from 3 μg of total RNA using a SuperScript III First-Strand Synthesis System (Invitrogen), Quantitative real-time PCR was performed by the ABI 7500 Fast system in a total of 20 μl of reaction containing 1 μl cDNA, 1 μl primer pairs, 8 μl ABI SYBR Green Master Mix (Applied Biosystems), and 8 μl RNAse-free water, and run at 95 °C, 3 min, followed by 40 cycles of 95 °C, 30 s and 60 °C, 30 s. Real-time PCR primer sequences are listed in Table 1.

### Western blot analysis

Proteins (30 μg) were resolved on a 4–12% Bis–Tris gradient SDS–PAGE under reducing conditions and transferred onto nitrocellulose membrane. The primary antibodies were RANKL, E-cadherin, vimentin, OPG, c-Met (Santa Cruz Biotechnology, Inc.), p-c-Met (Tyr-1230/34/35; Invitrogen), RANK (Amgen, Thousand Oaks, CA, USA), and N-cadherin (BD Transduction Laboratories, San Jose, CA, USA). AR (441), Chr-A (H-300), synaptophysin (SYP; D4), CD44 (DF1485), Sox-2 (Y-17), Nanog (5A10), LIN-28 (H-44) (Santa Cruz Biotechnology, Inc.), FOXA2 (D56D6; Cell Signaling Technology, Danvers, MA, USA), and PROM1 (CD133; Abnova, Taipei City, Taiwan) antibodies were used to detect neuroendocrine and stem cell differentiation. c-Myc (D84C12XP), Lamin A/C (Cell Signaling Technology), and Max (sc-197X) antibodies (Santa Cruz Biotechnology, Inc.) were used for nuclear protein detection.

### Immunohistochemistry

Human PCa tissue arrays in FFPE were purchased from Invitrogen and the PCa bone metastasis array (UWTMA22) was kindly provided by Dr Robert L Vessella from the University of Washington (Seattle, WA, USA). Immunohistochemical (IHC) staining followed a previously published protocol (Zhau *et al*. 2008) using primary antibodies against RANKL FL-317, c-Met C-12 (Santa Cruz Biotechnology, Inc.), and p-c-Met (pYpYpY1230/1234/1235; Invitrogen). IHC staining intensity was scored using the combined intensity and percentage of positive-scoring cells as previously reported (De Marzo *et al*. 1999). Strong intensity was scored as 3, intermediate as 2, weak as 1, and negative as 0. Each intensity score was then summed with the score of the percentage of cells that were stained, with >50% of the cells as 2, <50% of the cells as 1, and none as 0. One-way ANOVA was used to analyze the population scores between the two stages of cancer progression (bone vs benign, well-differentiated, or poorly-differentiated, or well-differentiated vs benign). The Gleason grade of the tumors and the quantification of the staining were determined by a pathologist, Dr Peizhen Hu.

### Construction of RANKL and c-Met promoter-luciferase reporter plasmid and deletion mutants

The 2.5 kb human RANKL promoter was amplified from the human BAC clone RP11-86N24 using primers 5′-GTGCACAGAATTCTTCAGGGGGCAAGTC-3′ (forward) and 5′-GCGAAGCTTCATGGCGCTCGGCCCTCTCG-3′ (reverse). The amplified fragment was subsequently subcloned into a firefly luciferase expression vector pGL3-basic (Promega). The authenticity of the 2.5 kb RANKL promoter region was confirmed by DNA sequencing. A series of deletion mutants of the RANKL promoter were generated from the 2.5 kb RANKL promoter using a QuikChange II XL Site-Directed Mutagenesis Kit following the manufacturer's instructions (Stratagene, Santa Clara, CA, USA). Human c-Met promoter-luciferase and its deletion constructs were provided by Dr Y Liu (Department of Pathology, University of Pittsburgh). Additional deletion constructs were also generated by the above protocol. Human AR promoter (6 kb) was provided by Dr Donald Tindall (Mayo Clinic).

### Transient transfection and luciferase reporter assay

RANKL, c-Met, and AR promoters and deletion mutants plus β-galactosidase plasmid (for transfection efficiency control) were transiently transfected into PCa cells using Lipofectamine 2000 (Invitrogen) for 48 h. After 48 h, the transfected cells were serum-starved for 24 h before treatment with RANKL, OPG, or RANKL plus OPG for another 48 h. For c-Myc inhibition, LNCaP cells were pretreated with 40 μM 10058-F4 c-Myc inhibitor for 4 h before treatment with RANKL. The luciferase promoter assay was conducted as described previously (Huang *et al*. 2010).

### Flow cytometry

Fluorescence-activated cell sorting (FACS) analyses were conducted as described previously (Sheridan *et al*. 2006). Stained cells were analyzed by BD Accuri C6 flow cytometer (BD, Franklin Lakes, NJ, USA) and results further analyzed using FlowJo Software (Ashland, OR, USA).

### Microarray analysis

Human U133plus2.0 array hybridizations were performed by the UCLA Clinical Microarray Core following the standard Affymetrix GeneChip Expression Analysis protocol. The acquisition of array images was undertaken using the Affymetrix GeneChip Command Console 1.1 (AGCC). The microarray data are publically available (GEO GSE48432).

### Computational analyses of global gene expression profile

The guanine cytosine robust multi-array analysis (GCMRA) method was applied to adjust the background signal. Probe intensities were normalized using the quantile normalization procedure (Wu *et al*. 2004). Integrated hypothesis testing included i) two independent tests, the *T*-test and the log2 median ratio test; ii) for each test, an empirical distribution of the null hypothesis that the means of the genes are not different was estimated by random permutations of the samples; iii) for each gene, the false discovery rate (FDR) was computed by a two-tailed test using the empirical distributions by Storey's method (Storey & Tibshirani 2003); and iv) the two sets of FDRs from the individual tests were combined to compute the overall FDR using Stouffer's method (Hwang *et al*. 2005). Finally, differentially expressed genes (DEGs) were selected for i) FDR <0.05 and ii) absolute expression changes larger than twofold. From 3345 DEGs (FDR <0.05, log2 fold change ≥1), we found that 1644 genes were upregulated and 1701 genes were significantly downregulated. A functional enrichment analysis of the list of up- and downregulated genes using the Database for Annotation, Visualization and Integrated Discovery (DAVID) Software (Bethesda, MD, USA) (Huang da *et al*. 2009) identified cellular processes enriched by the DEGs. Probability of significance was transformed into enrichment score, which is −log_10_ (enrichment *P* value).

### MR analysis for identification of key TFs

To identify key TFs, we first collected about 780 000 items of TF target interaction data for 391 TFs in the public databases including TRED (Zhao *et al*. 2005), EEDB (Severin *et al*. 2009), mSigDB (Subramanian *et al*. 2005), Amadeus (Linhart *et al*. 2008), OregAnno (Griffith *et al*. 2008), PAZAR (Portales-Casamar *et al*. 2007), and ChEA (Lachmann *et al*. 2010). We used Fisher's exact test (FET; Carro *et al*. 2010) to compute the significance of overlap between the TF-targets and DEGs of interest including RANK receptor downstream signaling, TF modules, EMT, stemness, and neuroendocrine differentiation. Then we selected eight TFs whose targets were significantly enriched by the upregulated DEGs (*P*<0.01 in FET).

### Identification of disease phenotypes

Upregulated genes in RANKL-overexpressed cells compared with control cells were used to identify associated human disease phenotypes using g:Profiler (Reimand *et al*. 2011), providing gene annotations from the HPO, a standardized vocabulary of phenotypic abnormalities encountered in human disease. Finally, we selected a list of disease/disease phenotype associations enriched by the 1644 upregulated genes with *P*<0.05.

### Statistical analysis

Differences between groups were analyzed using Student's *t*-test (two groups), one-way ANOVA (three or more groups), two-way ANOVA (two or more factors), or FET as appropriate. Where multiple groups were compared using ANOVA, a *post hoc* Tukey's or Dunnett's method was used to enable multiple comparisons between groups. Data that were not normally distributed were log-transformed before statistical tests were perfomed. A *P* value <0.05 was considered statistically significant. All statistical analysis was performed using R v2.15.1.

## Results

### RANKL expression increases with human PCa progression and is capable of driving non-metastatic PCa cells to colonize bone and soft tissues in mice

**Figure 1:**
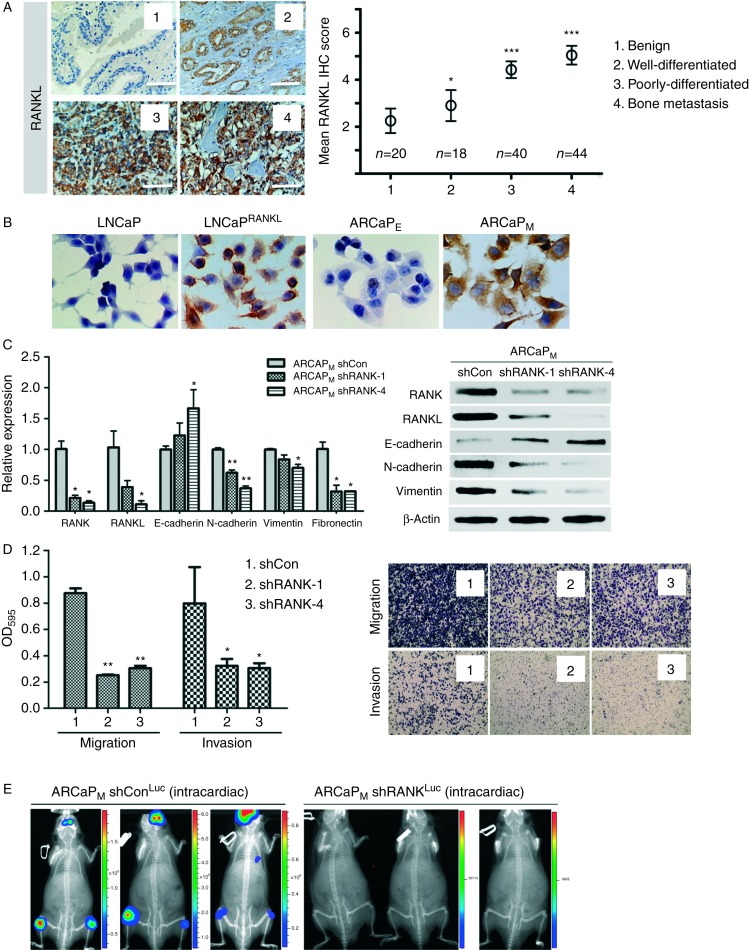
RANKL expression correlates with PCa progression in clinical specimens and cell models. (A) RANKL expression was negative or low in benign tumors but positive in well-differentiated and poorly-differentiated human primary and metastatic specimens. Increased RANKL expression correlated with human PCa progression from benign to bone metastasis. Relative scores of RANKL IHC staining in benign (*n*=20), well-differentiated (*n*=18), poorly-differentiated (*n*=40), and bone metastatic prostate tumors (*n*=44) are presented with statistical significance compared to benign tumors (400× magnification; ***P≤0.001; and *P≤0.05). (B) RANKL expression was shown to be negative or low in LN^Neo^ and ARCaP_E_ cells but high in LN^RANKL^ and ARCaP_M_ cells by immunohistochemical (IHC) staining (400×magnification). (C) ARCaP_M_ cells with RANK knockdown demonstrated decreased mesenchymal marker expression but increased epithelial marker expression detected by qRT-PCR and western blot analyses (*P<0.05; and **P<0.01). (D) RANK knockdown also decreased *in vitro* migration and invasion potential of ARCaP_M_ cells with the representative images of the migrated/invaded cells on the other side of the trans-wells, indicating a reversion of EMT to mesenchymal-to-epithelial transition (*P<0.05; and **P<0.01). (E) RANK knockdown in ARCaP_M_ cells failed to establish any metastasis in athymic nude mice (*n*=10; average total flux (photons/s): ARCaP_M_ shCon^Luc^ (intracardiac), 2.54×10^5^, 8.71×10^5^, and 2.01×10^5^; ARCaP_M_ shRANK^Luc^ (intracardiac), 3.45×10^4^ and 4.86×10^3^).

**Figure 2:**
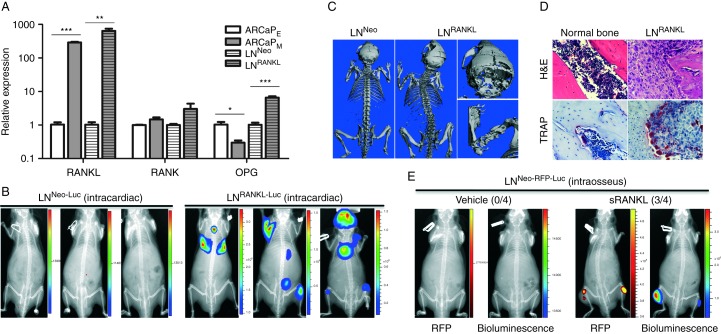
RANKL overexpression in PCa cells confers increased lethal and osteolytic bone metastasis in nude mice. (A) RANKL, RANK, and OPG expression was assessed in human PCa ARCaP_E_ and ARCaP_M_ EMT cell model and human LN^Neo^ and LN^RANKL^ cells by qRT-PCR (*P<0.05; **P<0.01; and ***P<0.001). (B) Representative bioluminescent images showed that luciferase-tagged LN^RANKL^ cells, but not luciferase-tagged LN^Neo^ control cells, induced bone and soft tissue metastases in nude mice (total flux (photons/s): LN^Neo-Luc^, 1.88×10^4^, 1.53×10^4^, and 1.05×10^4^; LN^RANKL-Luc^, 1.92×10^7^, 3.79×10^6^, and 4.14×10^8^). (C) Representative 3D μCT images of mice bearing LN^Neo^ and LN^RANKL^ cells showed that RANKL overexpression induced predominantly osteolytic lesions in mouse skeleton. (D) Representative H/E and positive TRAP staining in tumors in mouse bone induced by LN^RANKL^ cells. (E) Representative red fluorescent or bioluminescent images showed sRANKL (50 μg/kg i.p. twice a week, 2 weeks after intraosseous inoculation of 1×10^6^ PCa cells) induced tumor growth in mouse tibia inoculated with LN^Neo-RFP-Luc^ cells (*n*=4). No tumor was observed in vehicle-treated mice (average total radiant efficiency of RFP ((p/s)/(μW/cm^2^)) or total flux (p/s) of bioluminescence: vehicle, 3.68×10^8^ (RFP) and 8.96×10^3^ (bioluminescence); sRANKL, 2.34×10^9^ (RFP) and 8.63×10^6^ (bioluminescence)).

RANKL is prevalently expressed in human PCa specimens, with increased expression in higher grade and metastatic tumors compared with benign and low-grade PCa (Fig. 1A). We previously demonstrated that RANKL expression was significantly correlated with the overall survival of PCa patients (Hu *et al*. 2013). Moreover, RANKL expression correlates with the ability of PCa to metastasize to bone. ARCaP_M_ and LN^RANKL^ cells expressed respectively more intrinsic or transfected RANKL than their parental ARCaP_E_ and LN^Neo^ cells as determined by IHC (Fig. 1B). ARCaP_M_ cells were observed to express high levels of endogenous RANKL. Silencing of RANKL in ARCaP_M_ cells resulted in attenuation of the cells' mesenchymal phenotype (Fig. 1C) and their migratory and invasive potential (Fig. 1D). Consistent with this result, silencing of RANK, the cognate receptor for RANKL, abolished the cells' metastatic potential (Fig. 1E). Because androgen-responsive, non-metastatic LNCaP cells expressed low levels of RANKL, we tested whether stable, forced RANKL expression in LNCaP (LN^RANKL^) cells promoted RANKL–RANK signaling and bone metastasis. Relative expression levels of RANKL, RANK, and OPG in LN^RANKL^ and control neo-transfected LN^Neo^ cells, ARCaP_E_, and ARCaP_M_ are shown in Fig. 2A. To test the possibility that RANKL expression in PCa cells confers bone metastasis in mice, 1×10^6^ LN^RANKL^^-^^Luc^ or control LN^Neo-Luc^ cells were injected intracardially into mice. In marked contrast to the indolent LN^Neo-Luc^ (0/15), LN^RANKL-Luc^ cells metastasized primarily to bone (20/20) and, less uniformly, to lymph node (18/20), lung (8/20), and adrenal gland (17/20) as evaluated by bioluminescence (Fig. 2B). The predominant metastatic lesions in mouse skeletons were osteolytic, with a minor osteoblastic component as evaluated by μCT (Fig. 2C). The presence of tumor cells and the differentiation of pre-osteoclasts to mature osteoclasts in PCa bone lesions was validated by H/E and positive TRAP staining (Fig. 2D). Because LN^RANKL^ cells produced secreted RANKL (∼3 ng/ml; data not shown), we tested the effect of i.p. injection of recombinant soluble RANKL (sRANKL, 50 μg/kg, twice a week) on bone colonization of intratibially inoculated, non-metastatic LN^Neo-RFP-Luc^ cells. Mice that had received injections of RANKL exhibited bone metastases while no tumors formed in the skeleton in the animals that received injections of vehicle only (Fig. 2E). sRANKL was shown previously to induce bone turnover/resorption in rat bone; however, spontaneous tumor formation in response to this cytokine does not occur (McHugh *et al*. 2003).

### RANKL–RANK signaling alters an expansive gene expression program and controls c-Met and AR gene expression

**Figure 3:**
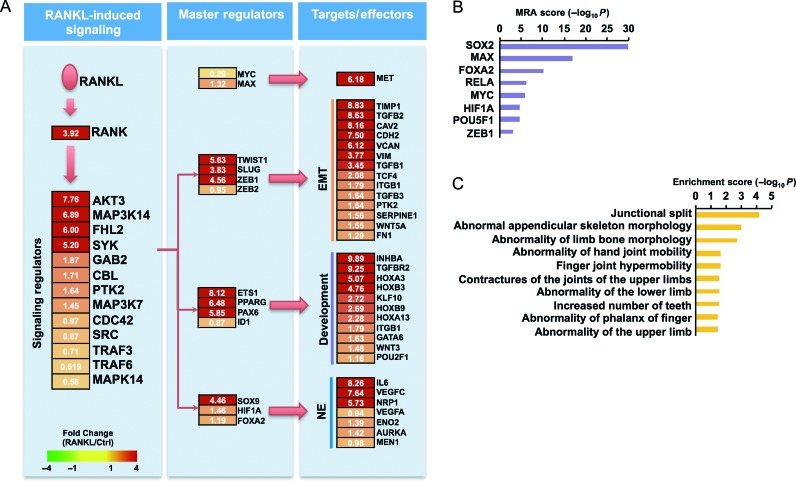
RANKL-perturbed network and master regulators. (A) RANKL pathway with upregulated genes in RANKL-overexpressing cells. RANKL-perturbed signaling and key TFs are shown. Node colors represent fold change in RANKL-overexpressing cells compared with controls. EMT, epithelial-to-mesenchymal transition; Development, development-related genes including differentiation and stemness; NE, neuroendocrine differentiation. (B) Independent analysis to identify master regulators predicted by master regulator analysis (MRA) score, which is −log_10_ transformed value of significance (*P* values). (C) Bioinformatics analysis using g:Profiler revealed that bone-related diseases are representative features of RANKL-overexpressing LNCaP cells.

We conducted a global analysis of RANKL-perturbed genes by transcriptome profiling of LN^RANKL^ and LN^Neo^ cells. This analysis showed that increased expression of RANKL promoted the expression of i) development-related genes regulating stem cells, neuronal differentiation, and morphogenesis; ii) genes controlling cell migration, angiogenesis, chemotaxis, and EMT; iii) genes associated with increased cell proliferation and decreased apoptosis; and iv) genes controlling bone development, bone morphogenesis, ossification, and tissue renewal (Supplementary Fig. 1, see section on supplementary data given at the end of this article). Increased RANKL expression significantly activated a number of cell signaling networks downstream from RANK that support cell growth, survival, and cell cycle progression, as well as TFs controlling growth factor and growth factor receptor signaling, EMT, and stem cell and neuroendocrine cell phenotypes (Fig. 3A). Key TFs in the RANKL-perturbed cell signaling network were identified by MR analysis using a computational approach described in the Supplementary Materials and Methods. We identified eight key TFs governing regulation of the 1644 RANKL-perturbed upregulated genes based on MR analysis (*P*<0.01; Fig. 3B). The targets of these eight key TFs accounted for 87% of genes upregulated in response to RANKL. Although expression of four of the key TFs, SOX2, MYC, POU5F1, and RELA, was not significantly different in the two cell lines, the number of their regulatory targets in the RANKL-perturbed cell signaling network (Fig. 3A) is highly significant according to MR analysis (Fig. 3B). Other key TFs also showed significant target occupation in RANKL-overexpressing cells compared with LN^Neo^ cells. RANKL was observed to activate a large number of genes and pathways closely associated with bone development and morphogenesis (Fig. 3C).

**Figure 4:**
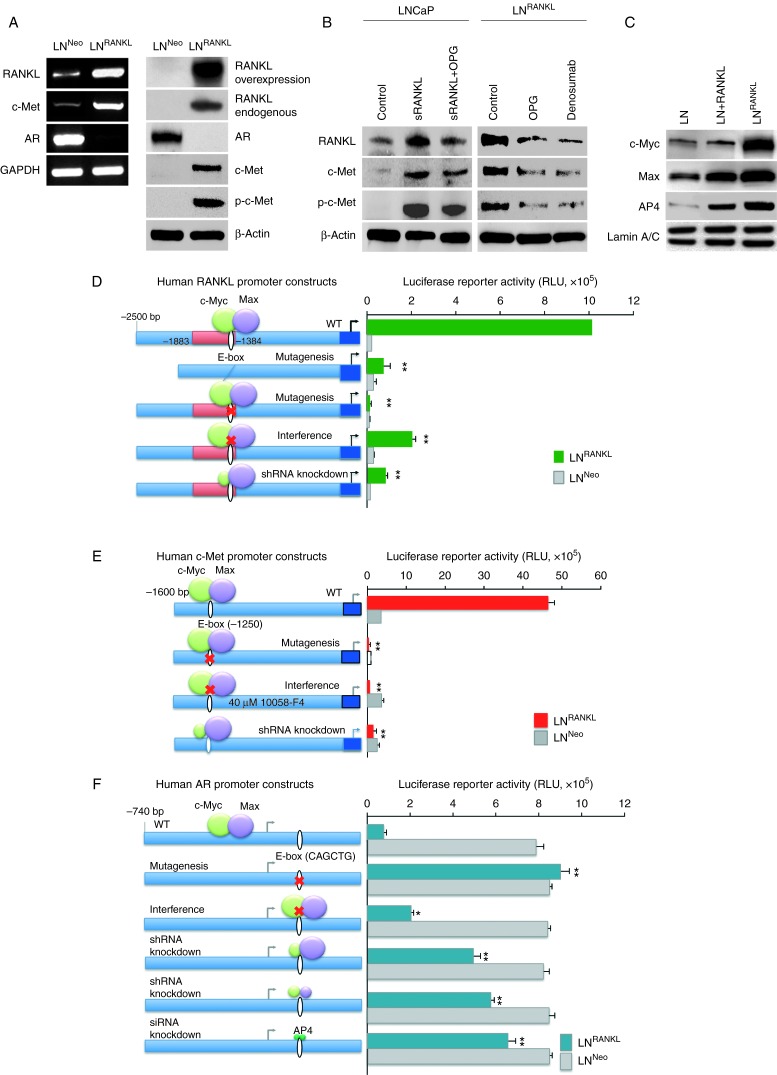
RANKL induces expression of RANKL and c-Met but suppresses AR expression/activation through c-Myc/Max and AP4 transcription factors in the LNCaP cell background. (A) RANKL overexpression upregulates endogenous RANKL and c-Met expression and c-Met phosphorylation but suppresses AR expression. (B) Exogenous RANKL treatment or intrinsic RANKL expression induces endogenous RANKL expression, c-Met expression, and c-Met phosphorylation. Inductions are partly sensitive to OPG or anti-RANKL antibody (denosumab) treatments. (C) RANKL–RANK signaling increases the level of c-Myc and Max expression. Increased c-Myc and Max nuclear proteins were observed in LNCaP cells treated with RANKL or in LN^RANKL^ cells as evaluated by western blot analysis. Lamin A/C was used as internal control. (D) Activities of human RANKL, (E) c-Met, and (F) AR promoter-luciferase reporter constructs were compared between LN^RANKL^ and LN^Neo^ cells. Site-directed mutagenesis was used to remove the E-box, the *cis*-element required for c-Myc/Max interaction. Complementary experiments confirmed the results by assaying promoter reporter activities under 10058-F4 (40 μM) exposure, known to inhibit c-Myc function by interference with its dimerization with Max, and by shRNA knockdown of the c-Myc, Max, or AP4 to suppress c-Myc/Max or AP4 binding to its E-box *cis*-element (*P<0.05; **P<0.01; and ***P<0.001).

LN^RANKL^ cells expressed increased endogenous RANKL and c-Met and decreased AR mRNA and protein (Fig. 4A). c-Met activation, as indicated by increased phosphorylation (at tyrosine 1230/1234/1235 sites), was also increased in LN^RANKL^ cells. Expression of RANKL, c-Met, and activated c-Met was attenuated by a RANKL decoy receptor, OPG, or an anti-RANKL antibody, denosumab (Fig. 4B). These findings indicate that RANK–RANKL signaling significantly alters an oncogenic signaling program. To approach the mechanism underlying these changes, we used RANKL-, c-Met-, and AR-promoter luciferase constructs to identify *cis*-elements and TFs that may control them. Both the RANKL- and c-Met promoter regions contain E boxes (CACGTG) at −1384 or −1250 bp respectively. These are consensus regions for c-Myc/Max binding (Fig. 4D and E). Upregulation of RANKL and c-Met expression by enhanced RANK–RANKL signaling was mediated through direct interaction of the c-Myc/Max heterodimer to the E-box region within RANKL and c-Met promoters as assessed by mutation (Fig. 4D and E), chromatin immunoprecipitation (ChIP) (Supplementary Fig. 2A and C, see section on supplementary data given at the end of this article), and electrophoretic mobility shift assay (EMSA) (Supplementary Fig. 2B). Deletion of the *cis*-elements required for c-Myc/Max interaction dramatically decreased promoter-luciferase activities in LN^RANKL^ cells (Fig. 4D and E). Similar downregulation of promoter activity was observed when LN^RANKL^ cells were treated pharmacologically with 10058-F4 (40 μM), which interferes with c-Myc function by preventing its dimerization with Max, or by lentiviral c-Myc shRNA knockdown, which greatly depressed c-Myc expression and hindered c-Myc/Max binding to its *cis*-element. The feed-forward action of RANKL–RANK signaling in promoting RANKL and c-Met via increased c-Myc/Max TF protein expression found in LN^RANKL^ was also observed in LNCaP cells treated with exogenous recombinant RANKL (Fig. 4C). Previously, Nadiminty *et al*. (2012) reported that AR transcription is regulated by miRNA-let7c via c-Myc. Interestingly, we observed coordinated suppression of AR expression, mediated through c-Myc/Max heterodimer and/or AP4 homodimer, and AP4 has been shown to be a conserved and direct target gene of c-Myc (Cole & McMahon 1999, Jung *et al*. 2008). The interaction site was identified at the E-box/AP4 binding site (CAGCTG) on the AR promoter region at +374 bp. Deletion of the AP4 site significantly upregulated AR promoter activity in LN^RANKL^ cells. Similarly, interference with c-Myc and Max dimerization with 10058-F4 or genetic ablation of c-Myc, c-Myc and Max, or AP4 using shRNA or siRNA also increased AR promoter activity in LN^RANKL^ cells (Fig. 4F). ChIP analysis further demonstrated direct interaction of c-Myc, Max, and AP4 with the AP4 site within the AR promoter (Supplementary Fig. 2D). AR expression at the RNA and protein level was restored by knocking down c-Myc, c-Myc and Max, or AP4 in LN^RANKL^ cells (Supplementary Fig. 3, see section on supplementary data given at the end of this article).

### RANKL–RANK signaling promotes EMT and stem and neuroendocrine phenotypes

Consistent with our previous report using ARCaP cells (Zhau *et al*. 2008), RANKL–RANK signaling also promoted the expression of EMT markers in LNCaP cells. Increased expression of RANKL induced the expression of mesenchymal features, including subtle morphological changes to a more spreading morphology (Supplementary Fig. 4A, see section on supplementary data given at the end of this article), mesenchymal gene expression profiles (increased N-cadherin, and vimentin expression but decreased E-cadherin expression, Supplementary Fig. 4B), and more aggressive cell behaviors (increased cell motility, migration, invasion, and anchorage-independent growth, Supplementary Fig. 4C and D). LNCaP cell-derived RANKL or exogenously administered recombinant RANKL are biologically functional and induce pre-osteoclast maturation as measured by increased TRAP+ staining, when co-cultured with mouse RAW264.7 pre-osteoclasts (Supplementary Fig. 5, see section on supplementary data given at the end of this article). This activity was attenuated by a RANKL decoy receptor, OPG.

**Figure 5:**
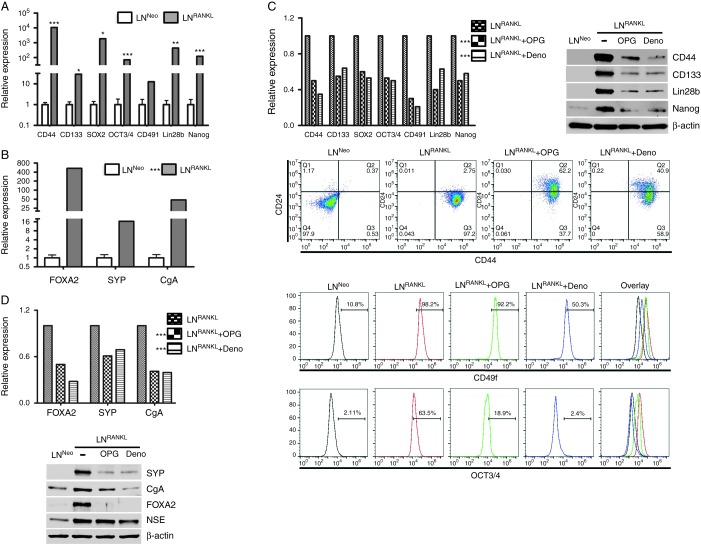
RANKL promotes stem cell and neuroendocrine properties of PCa cells. LN^RANKL^ cells have elevated gene expression of (A) stem cell markers and (B) neuroendocrine markers confirmed by qRT-PCR. (C) Upregulation of stem cell markers by RANKL expression can be attenuated by OPG and denosumab treatments as analyzed by qRT-PCR, western blot, and flow cytometry. (D) OPG and denosumab also downregulate the expression of neuroendocrine markers in LN^RANKL^ cells, confirmed by both qRT-PCR and western blot analyses. (B, C and D) Statistical significance of differences between the treatment groups and the control group (LN^RANKL^) were calculated based on a global comparison for the set of genes. *P<0.05, **P<0.01, and ***P<0.001.

RANKL–RANK signaling also promoted stem cell and neuroendocrine differentiation in LNCaP cells. Figure 5A shows increased expression of the stem cell markers CD44, CD133, SOX2, OCT3/4, CD49f, Lin28b, and Nanog. Figure 5B shows elevated expression of the neuroendocrine markers FOXA2, SYP, and chromogranin A. Induced expression of stem cell and neuroendocrine markers was partly blocked by denosumab and OPG, indicating that the maintenance of these phenotypes requires continuous RANKL–RANK signaling (Fig. 5C and D). Consistent with higher intrinsic RANKL expressed by ARCaP_M_ cells, we also observed that this cell line expressed higher levels of TFs that regulate EMT, stem, and neuroendocrine phenotypes (Supplementary Fig. 6, see section on supplementary data given at the end of this article). Together, these results are consistent with previous reports showing that cancer cells undergoing EMT expressed stem cell characteristics (Kong *et al*. 2011). The emergence of an neuroendocrine phenotype in the LNCaP as well as ARCaP_M_ cell backgrounds in response to RANKL–RANK signaling has not been reported and is probably due to the activation of TFs such as FoxA2, SOX9, and HIF1α that regulate the neuroendocrine phenotype of PCa cells (Qi *et al*. 2010).

### RANK, c-Met, and c-Myc/Max are required for bone colonization by LN^RANKL^ cells

**Figure 6:**
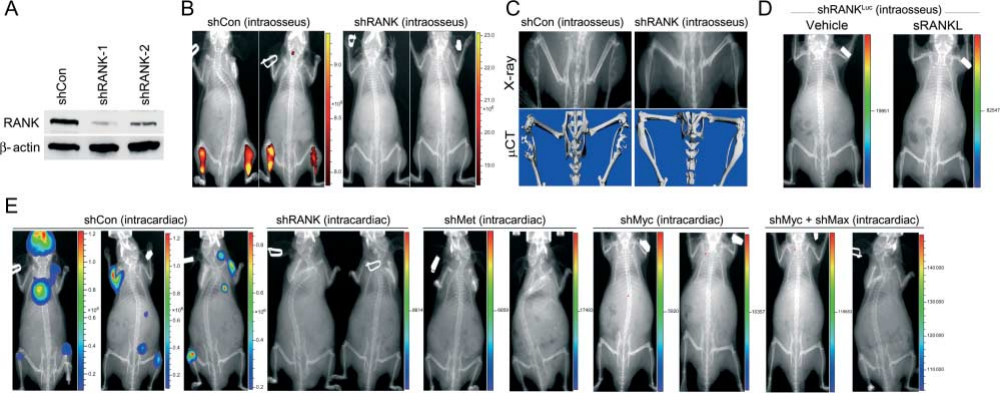
Abrogation of RANK or its downstream target transcription factors, c-Myc/Max, or its effector/target, c-Met abolishes the metastatic potential of LN^RANKL^ cells. (A) Partial RANK knockdown in LN^RANKL^ cells was confirmed by western blot analysis. (B) Representative NIR fluorescent images showed that RANK-knocked-down LN^RANKL^ cells (*n*=10) form no or very small tumors undetectable by NIR fluorescent imaging, but LN^RANKL^ cells transduced with control shRNA (shCon) (*n*=10) form obvious intratibial tumors (average total radiant efficiency of NIR ((p/s)/(μW/cm^2^)): shCon (intraosseus), 4.86×10^10^ and 3.37×10^10^; shRANK (intraosseus), 2.63×10^5^ and 3.84×10^5^). (C) X-ray and μCT scans showed that LN^RANKL^ cells induced osteolytic lesions in mouse tibia, unlike RANK-knocked-down LN^RANKL^ cells where mouse tibia were intact with minimal detectable bone lesions. (D) Representative *in vivo* bioluminescent images further demonstrated that administration of recombinant sRANKL (50 μg/kg s.c. twice a week, 2 weeks after intraosseous inoculation of test cell line) fails to induce intratibial tumor formation of luciferase-tagged RANK-knocked down LN^RANKL^ cells in mice (*n*=10) (total flux (photons/s): shRANK-1 (intraosseus) vehicle, 2.53×10^3^ and shRANK-1 (intraosseus)+sRANKL, 1.80×10^4^). (E) Representative *in vivo* bioluminescent images further demonstrated that luciferase-tagged LN^RANKL^ shCon cells, but not luciferase-tagged LN^RANKL^ cells with RANK (*n*=10), c-Met (*n*=10), c-Myc (*n*=8), or c-Myc/Max (*n*=8) knockdown, induce bone and soft tissue metastases after intracardiac injection (average total flux (photons/s): shCon (intracardiac), 4.60×10^8^, 9.71×10^6^, and 4.01×10^7^; shRANK (intracardiac), 4.27×10^3^ and 6.13×10^3^; shMet (intracardiac), 4.52×10^3^ and 2.25×10^4^; shMyc (intracardiac), 8.51×10^3^ and 1.83×10^4^; and shMyc+shMax (intracardiac), 8.65×10^4^ and 1.64×10^5^).

RANKL–RANK signaling seems obligatory for bone colonization. LN^RANKL^ cells with RANK knockdown (Fig. 6A) formed very small tumors in mice tibias, undetectable by NIR dye-based fluorescence imaging (Yang *et al*. 2010) but detectable by X-ray (Fig. 6B); moreover, the tumors were less osteolytic and much less bone resorptive compared with bone tumors derived from control shRNA-transduced LN^RANKL^ cells (Fig. 6C). Administration of sRANKL failed to induce bone tumor formation in mice bearing intratibially injected RANK-silenced LN^RANKL^ cells (Fig. 6D). Knockdown of the RANK downstream target TFs, c-Myc/Max, or its effector/target, c-Met, completely abolished the ability of LN^RANKL^ cells to form skeletal or soft tissue metastasis in mice (Fig. 6E). These results support the obligatory role of RANKL–RANK, c-Myc/Max, and c-Met signaling in cancer bone colonization.

### Metastatic LN^RANKL^ cells transform neighboring non-metastatic LN^Neo^ cells

**Figure 7:**
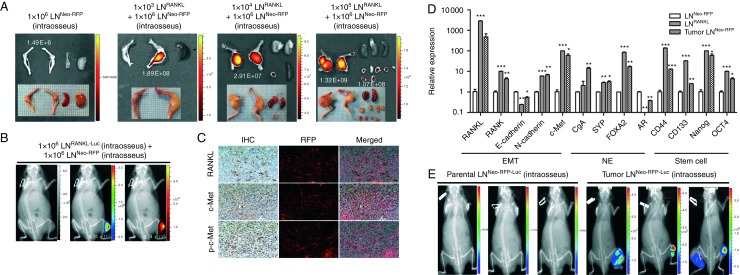
Metastatic LN^RANKL^ cells can transform neighboring non-metastatic LN^Neo^ cells to undergo EMT, express neuroendocrine (NE) and stem cell biomarkers, and form tumors in mouse skeleton. (A) A small population of LN^RANKL^ cells was capable of recruiting non-metastatic LN^Neo-RFP^ cells, which were incapable of forming tumors by themselves, to participate in bone colonization in mice. One thousand, ten thousand, and one hundred thousand LN^RANKL^ cells were co-inoculated with one million LN^Neo-RFP^ cells in both tibia of nude mice. Tibial tumors and tumors disseminated from bone to soft tissues were harvested and subjected to fluorescent imaging to detect the red fluorescent signal from non-metastatic LN^Neo-RFP^ cells using the Xenogen imaging system at an excitation of 535 nm and emission of 620 nm. Spleens were also harvested and used as negative controls. Total radiant efficiency ((p/s)/(μW/cm^2^)) of the RFP in tumors was labeled in white in the corresponding tumors. (B) Representative *in vivo* bioluminescent and red fluorescent images were also demonstrated with mice bearing intratibial inoculation of either luciferase-tagged LN^RANKL^ cells followed by intracardiac inoculation of LN^Neo-RFP^ cells to test the homing potential of RFP-tagged LNCaP^Neo^ cells. (C) IHC and fluorescence images were obtained from chimeric tumors induced in mouse skeleton by inoculating 1000 LN^RANKL^ cells plus 1×10^6^ LN^Neo-RFP^ cells. Representative IHC and fluorescence images of the tumors were merged (200× magnification). Data show co-localization of RFP cells with RANKL, c-Met, and p-c-Met expression in prostate tumors from mouse skeleton. (D) LN^Neo-RFP^ cells harvested from chimeric tumors (tumor LN^Neo-RFP^) acquired EMT, neuroendocrine, and stem cell properties demonstrated by relative expression of markers detected by qRT-PCR (*P<0.05; **P<0.01; and ***P<0.001). (E) Representative bioluminescent images demonstrated tibial tumor formation induced by luciferase-tagged tumor-derived LN^Neo-RFP^ cells but not by luciferase-tagged parental LN^Neo-RFP^ cells (total flux (photons/s): parental LN^Neo-RFP-Luc^, 5.77×10^3^; 2.09×10^4^; and 1.56×10^4^ and tumor LN^Neo-RFP-Luc^, 5.35×10^6^; 9.94×10^5^; and 8.52×10^5^).

We tested the ability of 1×10^3^ LN^RANKL^ cells combined with 1×10^6^ non-metastatic 1×10^6^ LN^Neo^ cells to form tumors in mouse skeleton after intratibial injection. While neither LN^RANKL^ nor LN^Neo^ cells when injected individually in these amounts form tumors during a 3-month observation period, tumors arose from the mixed cell populations in <2 months (Fig. 7A). A similar result was observed when LN^RANKL^ cells were injected into the tibia and LN^Neo-RFP^ cells were injected by the intracardiac route (Fig. 7B). IHC analysis revealed that tumors were chimeras consisting of RFP-positive and -negative cells that abundantly expressed RANKL, c-Met, and p-c-Met (Fig. 7C), although parental LN^Neo-RFP^ cells expressed undetectable levels of RANKL, c-Met, and p-c-Met in culture. We cloned LN^Neo-RFP^ cells from harvested chimeric tumors using the RFP marker and characterized the gene expression profiles of the cultured sublines. Figure 7D shows that tumor-derived LN^Neo-RFP^ cells in culture exhibited patterns of gene expression resembling LN^RANKL^ cells, patterns distinctly different from those of the parental LN^Neo-RFP^ cells. We subsequently labeled both parental and tumor-derived LN^Neo-RFP^ cells with luciferase, and when injected intratibially, only the tumor-derived LN^Neo-RFP-Luc^ cells but not the parental LN^Neo-RFP-Luc^ cells colonized the mouse skeleton (Fig. 7E). These results, in aggregate, indicate that LN^RANKL^ cells are able to transform or reprogram neighboring bystander LN^Neo-RFP^ cells to generate tumors in bone.

In summary, these experiments demonstrate that a RANK-mediated signaling network can drive PCa tumor growth in the mouse skeleton. This mechanism involves a feed-forward process in which RANKL and c-Met are induced, and AR is downregulated, as well as a transcriptional network involving c-Myc/Max as an essential node. In this model system, downregulation of RANK, c-Myc/Max, or c-Met expression abolished the bone-homing potential in mice. RANKL-expressing PCa cells were also shown to exhibit transforming potential by altering the gene expression of neighboring bystander cells and coopting them to participate in bone colonization.

## Discussion

Hormone-refractory, bone metastatic human prostate and breast cancers are typically lethal; however, little is known about the intrinsic and extrinsic factors that contribute to tumor colonization of the skeleton. Previous reports identified a potential role for VCAM1–integrin interaction (Lu *et al*. 2011), hematopoietic stem cells (Park *et al*. 2011), immunoregulatory T-cells (Tan *et al*. 2011), cancer-associated fibroblasts or macrophages (Paland *et al*. 2009, Qian *et al*. 2011), and soluble protein factors like HGF, bone morphogenic proteins, insulin-like growth factors, the bone TF RUNX2 (Akech *et al*. 2010, Gherardi *et al*. 2012, Hiraga *et al*. 2012), and microRNAs (Cai *et al*. 2013) in cancer metastasis. The pathophysiological roles of these factors and cell types in bone metastasis, however, are largely correlational and have not been demonstrated in animal models. Our study is the first, to our knowledge, to show that RANKL, derived either from tumor cells or delivered as recombinant protein, has the ability to promote cancer bone and soft tissue colonization through RANK-mediated signal amplification. The following salient features of RANKL–RANK signal amplification were observed: i) transcriptional activation of RANKL and c-Met coincident with attenuation of AR expression. This is attributed to the highly coordinated induction of common TF complexes, c-Myc/Max and AP4 (Fig. 4). ii) PCa bone colonization can be initiated by a small number of RANKL-expressing bone-colonizing metastasis-initiating cells that can recruit non-metastatic bystander cells to participate (Fig. 7A and B). iii) This recruitment of bystander cells involves feed-forward signal amplification, involving increased expression of RANKL, RANK, and c-Met (Fig. 7C and D). We suggest that the continued maintenance of RANK signaling output in the recruited bystander cells could be responsible for their tumorigenicity, increasing cancer cell growth, survival, and angiogenesis by activating p-c-Met and promoting EMT, stem cell, and neuroendocrine cell differentiation (Fig. 5).

Response to tumor-derived RANKL or exposure to recombinant RANKL protein, LN^RANKL^, or LN^Neo^/LN^RFP^ cells in the bone microenvironment may establish a premetastatic niche through three potential mechanisms: i) interaction with cancer cells to amplify downstream targets and effectors through upregulation of a host of MR TFs, such as c-Myc/Max, FoxA2, Sox2, Sox9, Oct4, NF-κB RelA, HIF1α, and Zeb1, that promote growth, survival, angiogenesis, EMT and stem and neuroendocrine-cell phenotypes that promote cancer cell growth in bone (Fig. 3). ii) The effects of RANKL-expressing cells on bystander cells appear permanent, based on our *ex vivo* culture and profiling of RFP-labeled cells derived from bone metastases. These results support our previous observation that human PCa cells inoculated into immunodeficient mice recruited bystander cells, which underwent genetic modification, as exhibited by their cytogenetic profiles (Pathak *et al*. 1997). iii) Interaction with pre-osteoclasts promotes osteoclastogenesis, increases bone turnover, and releases soluble and insoluble factors from the skeleton, supporting cancer growth and survival in bone. Our data collectively indicate that autocrine and paracrine RANKL–RANK signaling is critical for the development of bone and soft tissue metastases in mice.

RANKL is expressed by inflammatory T and B cells, osteoblasts, marrow stromal cells, mesenchymal stem cells, and stromal fibroblasts (Thomas *et al*. 2001). RANKL expression can be regulated by endocrine factors and dietary cholesterol (Sanbe *et al*. 2009). The functional roles of RANKL depend on the triad relationship of RANKL, RANK, and OPG where RANKL activity is modulated by the presence of RANK receptor and OPG (Ando *et al*. 2008). The pathophysiological significance of our findings is that RANKL expression is affected by inflammation and significantly augmented by hormonal deprivation (Li *et al*. 2009) or increased dietary cholesterol (Nuche-Berenguer *et al*. 2011). Interestingly, cancer bone and soft tissue dissemination is also known to be increased by inflammatory infiltrates in tumors. Increased cancer metastases were also observed in patients with higher dietary cholesterol and hosts undergoing androgen-deprivation therapy (Tan *et al*. 2011).

Coordinated cell signal network expansion downstream from RANKL–RANK in PCa cells is associated with the invasive phenotype that commonly develops in CRPC patients upon disease progression or under hormonal or chemotherapeutic intervention (Mulholland *et al*. 2012). Our current model suggests that the RANKL–RANK-mediated signal network downregulates AR and renders the PCa cells more aggressive and metastatic, including the ability to recruit and transform non-metastatic bystander cells. While the exact mechanisms by which AR downregulation promotes PCa aggressiveness is unknown, it has however been observed that approximately 25% of CRPC patients actually lose AR expression and develop rapid progressive bone metastasis. These patients express low levels of AR and PSA but have been observed to exhibit explosive metastatic disease clinically. Our study raises several important questions that require further study in clinical settings. Can LN^RANKL^ cells be identified in the primary, at metastatic sites, or in the systemic circulation and be selectively or more effectively targeted based on their unique phenotype? As androgen deprivation can induce PCa stem cell and neuroendocrine differentiation, can androgen deprivation also induce the resurgence of PCa cells with a phenotype resembling LN^RANKL^? Can the dietary cholesterol, hormonal or inflammatory status of patients be modulated to reduce overall levels of RANKL or RANKL–RANK signaling, to minimize the tumor cell population that initiates PCa metastasis, thus reducing tumor recurrence and distant dissemination? A better understanding of RANKL–RANK signaling in clinical cancer metastasis will improve cancer bone targeting and patient survival.

In summary, our results indicate that RANKL, either derived from the tumor or from the host, plays a key role in cancer bone metastasis. A small population of LN^RANKL^ cells was observed to initiate and promote cancer bone and soft tissue metastases by recruiting bystander cells to form tumors in bone. The mechanism underlying this recruitment appears to involve a feed-forward mechanism in which RANKL, RANK, and c-Met expression is increased and AR is downregulated. RANKL alters a large transcriptional program that appears to govern formation of the premetastatic niche as well as emergence of osteomimetic, EMT, and stem and neuroendocrine differentiation. Our results raise a new paradigm beyond the clonal expansion and evolution of cancer cells, where a population of metastasis-initiating PCa cells recruits and activates bystander cells, including ‘dormant’ cancer cells, through RANK-mediated signal amplification.

## Supplementary data

This is linked to the online version of the paper at http://dx.doi.org/10.1530/ERC-13-0548.

## Figures and Tables

**Table 1:** Real-time PCR primer sequences

**Genes**	**Real-time PCR primer sequences**
*RANKL*	F: 5′-TCCCATCTGGTTCCCATAAA-3′
	R: 5′-GGTGCTTCCTCCTTTCATCA-3′
*RANK*	F: 5′-GTCTGGAAGCTCCCCTGGT-3′
	R: 5′-TTGAAGTTCATCACCTGCCC-3′
*OPG*	F: 5′-TTCCGGAAACAGTGAATCAA-3′
	R: 5′-CGCTGTTTTCACAGAGGTCA-3′
*E-cadherin*	F: 5′-CCCGGGACAACGTTTATTAC-3′
	R: 5′-GCTGGCTCAAGTCAAAGTCC-3′
*N-cadherin*	F: 5′-GGTGGAGGAGAAGAAGACCAG-3′
	R: 5′-GGCATCAGGCTCCACAGT-3′
*Vimentin*	F: 5′-GGAAGAGAACTTTGCCGTTGAA-3′
	R: 5′-GTGACGAGCCATTTCCTCCTT-3′
*c-Myc*	F: 5′-TCAAGAGGCGAACACACAAC-3′
	R: 5′-GGCCTTTTCATTGTTTTCCA-3′
*c-Met*	F: 5′-TGAAATTCATCCAACCAAATCTT-3′
	R: 5′-AATAGAAAACTGACAATGTTGAGAGG-3′
*CD44*	F: 5′-TCAGAGGAGTAGGAGAGAGGAAAC-3′
	R: 5′-GAAAAGTCAAAGTAACAATAACAGTGG-3′
*CD133*	F: 5′-CAGAGTACAACGCCAAACCA-3′
	R: 5′-AAATCACGATGAGGGTCAGC-3′
*CD49f*	F: 5′-TTGGAGCTCCGTATGATGACTTGG-3′
	R: 5′-GGATCTCCACTGAGGCAGTTATGG-3′
*OCT4*	F: 5′-AGCAAAACCCGGAGGAGT-3′
	R: 5′-CCACATCGGCCTGTGTATATC-3′
*SOX2*	F: 5′-TTGCTGCCTCTTTAAGACTAGGA-3′
	R: 5′-CTGGGGCTCAAACTTCTCTC-3′
*Nanog*	F: 5′-ATGCCTCACACGGAGACTGT-3′
	R: 5′-AAGTGGGTTGTTTGCCTTTG-3′
*Lin28b*	F: 5′-CTGTCAGAGCATCATGCACATG-3′
	R: 5′-GGGTGGCTGTGCAACATTTT-3′
*SYP*	F: 5′-TGCGCTAGAGCATTCTGGG-3′
	R 5′-CTTAAAGCCCTGGCCCCTTCT-3′
*CgA*	F: 5′-CCCCACTGTAGTGCTGAACC-3′
	R: 5′-GGAGTGCTCCTGTTCTCCC-3′
*FOXA2*	F: 5′-TCTTAAGAAGACGACGGCTTCAG-3′
	R: 5′-TTGCTCTCTCACTTGTCCTCGAT-3′
*SOX2*	F: 5′-TTGCTGCCTCTTTAAGACTAGGA-3′
	R: 5′-CTGGGGCTCAAACTTCTCTC-3′
*AR*	F: 5′-GACCAGATGGCTGTCATTCA-3′
	R: 5′-GGAGCCATCCAAACTCTTGA-3′

## References

[bib1] Akech J, Wixted JJ, Bedard K, van der Deen M, Hussain S, Guise TA, van Wijnen AJ, Stein JL, Languino LR, Altieri DC (2010). Runx2 association with progression of prostate cancer in patients: mechanisms mediating bone osteolysis and osteoblastic metastatic lesions. Oncogene.

[bib2] Ando K, Mori K, Redini F, Heymann D (2008). RANKL/RANK/OPG: key therapeutic target in bone oncology. Current Drug Discovery Technologies.

[bib3] Birchmeier C, Birchmeier W, Gherardi E, Vande Woude GF (2003). Met, metastasis, motility and more. Nature Reviews. Molecular Cell Biology.

[bib4] Cai J, Guan H, Fang L, Yang Y, Zhu X, Yuan J, Wu J, Li M (2013). MicroRNA-374a activates Wnt/β-catenin signaling to promote breast cancer metastasis. Journal of Clinical Investigation.

[bib5] Carro MS, Lim WK, Alvarez MJ, Bollo RJ, Zhao X, Snyder EY, Sulman EP, Anne SL, Doetsch F, Colman H (2010). The transcriptional network for mesenchymal transformation of brain tumours. Nature.

[bib6] Cole MD, McMahon SB (1999). The Myc oncoprotein: a critical evaluation of transactivation and target gene regulation. Oncogene.

[bib7] Coleman RE (2001). Metastatic bone disease: clinical features, pathophysiology and treatment strategies. Cancer Treatment Reviews.

[bib8] De Marzo AM, Knudsen B, Chan-Tack K, Epstein JI (1999). E-cadherin expression as a marker of tumor aggressiveness in routinely processed radical prostatectomy specimens. Urology.

[bib9] Dougall WC (2011). Molecular pathways: osteoclast-dependent and osteoclast-independent roles of the RANKL/RANK/OPG pathway in tumorigenesis and metastasis. Clinical Cancer Research.

[bib10] Gherardi E, Birchmeier W, Birchmeier C, Vande Woude G (2012). Targeting MET in cancer: rationale and progress. Nature Reviews. Cancer.

[bib11] Griffith OL, Montgomery SB, Bernier B, Chu B, Kasaian K, Aerts S, Mahony S, Sleumer MC, Bilenky M, Haeussler M (2008). ORegAnno: an open-access community-driven resource for regulatory annotation. Nucleic Acids Research.

[bib12] Hanada R, Leibbrandt A, Hanada T, Kitaoka S, Furuyashiki T, Fujihara H, Trichereau J, Paolino M, Qadri F, Plehm R (2009). Central control of fever and female body temperature by RANKL/RANK. Nature.

[bib13] Hiraga T, Myoui A, Hashimoto N, Sasaki A, Hata K, Morita Y, Yoshikawa H, Rosen CJ, Mundy GR, Yoneda T (2012). Bone-derived IGF mediates crosstalk between bone and breast cancer cells in bony metastases. Cancer Research.

[bib14] Hu P, Chung LW, Berel D, Frierson HF, Yang H, Liu C, Wang R, Li Q, Rogatko A, Zhau HE (2013). Convergent RANK- and c-Met-mediated signaling components predict survival of patients with prostate cancer: an interracial comparative study. PLoS ONE.

[bib15] Huang WC, Wu D, Xie Z, Zhau HE, Nomura T, Zayzafoon M, Pohl J, Hsieh CL, Weitzmann MN, Farach-Carson MC (2006). β2-Microglobulin is a signaling and growth-promoting factor for human prostate cancer bone metastasis. Cancer Research.

[bib16] Huang WC, Zhau HE, Chung LW (2010). Androgen receptor survival signaling is blocked by anti-β2-microglobulin monoclonal antibody via a MAPK/lipogenic pathway in human prostate cancer cells. Journal of Biological Chemistry.

[bib17] Huang da W, Sherman BT, Lempicki RA (2009). Systematic and integrative analysis of large gene lists using DAVID bioinformatics resources. Nature Protocols.

[bib18] Hwang D, Rust AG, Ramsey S, Smith JJ, Leslie DM, Weston AD, de Atauri P, Aitchison JD, Hood L, Siegel AF (2005). A data integration methodology for systems biology. PNAS.

[bib19] Josson S, Nomura T, Lin JT, Huang WC, Wu D, Zhau HE, Zayzafoon M, Weizmann MN, Gururajan M, Chung LW (2011). β2-Microglobulin induces epithelial to mesenchymal transition and confers cancer lethality and bone metastasis in human cancer cells. Cancer Research.

[bib20] Jung P, Menssen A, Mayr D, Hermeking H (2008). AP4 encodes a c-MYC-inducible repressor of *p21*. PNAS.

[bib21] Knudsen BS, Edlund M (2004). Prostate cancer and the met hepatocyte growth factor receptor. Advances in Cancer Research.

[bib22] Kong D, Li Y, Wang Z, Sarkar FH (2011). Cancer stem cells and epithelial-to-mesenchymal transition (EMT)-phenotypic cells: are they cousins or twins?. Cancer.

[bib23] Lachmann A, Xu H, Krishnan J, Berger SI, Mazloom AR, Ma'ayan A (2010). ChEA: transcription factor regulation inferred from integrating genome-wide ChIP-X experiments. Bioinformatics.

[bib24] van Leenders GJ, Sookhlall R, Teubel WJ, de Ridder CM, Reneman S, Sacchetti A, Vissers KJ, van Weerden W, Jenster G (2011). Activation of c-MET induces a stem-like phenotype in human prostate cancer. PLoS ONE.

[bib25] Li X, Ominsky MS, Stolina M, Warmington KS, Geng Z, Niu QT, Asuncion FJ, Tan HL, Grisanti M, Dwyer D (2009). Increased RANK ligand in bone marrow of orchiectomized rats and prevention of their bone loss by the RANK ligand inhibitor osteoprotegerin. Bone.

[bib26] Linhart C, Halperin Y, Shamir R (2008). Transcription factor and microRNA motif discovery: the Amadeus platform and a compendium of metazoan target sets. Genome Research.

[bib27] Lu X, Mu E, Wei Y, Riethdorf S, Yang Q, Yuan M, Yan J, Hua Y, Tiede BJ, Lu X (2011). VCAM-1 promotes osteolytic expansion of indolent bone micrometastasis of breast cancer by engaging α4β1-positive osteoclast progenitors. Cancer Cell.

[bib28] McHugh NA, Vercesi HM, Egan RW, Hey JA (2003). Receptor activator of NF-κB ligand arrests bone growth and promotes cortical bone resorption in growing rats. Journal of Applied Physiology.

[bib29] Mulholland DJ, Kobayashi N, Ruscetti M, Zhi A, Tran LM, Huang J, Gleave M, Wu H (2012). *Pten* loss and RAS/MAPK activation cooperate to promote EMT and metastasis initiated from prostate cancer stem/progenitor cells. Cancer Research.

[bib30] Nadiminty N, Tummala R, Lou W, Zhu Y, Zhang J, Chen X, eVere White RW, Kung HJ, Evans CP, Gao AC (2012). MicroRNA let-7c suppresses androgen receptor expression and activity via regulation of Myc expression in prostate cancer cells. Journal of Biological Chemistry.

[bib31] Nuche-Berenguer B, Lozano D, Gutierrez-Rojas I, Moreno P, Marinoso ML, Esbrit P, Villanueva-Penacarrillo ML (2011). GLP-1 and exendin-4 can reverse hyperlipidic-related osteopenia. Journal of Endocrinology.

[bib32] Odero-Marah VA, Wang R, Chu G, Zayzafoon M, Xu J, Shi C, Marshall FF, Zhau HE, Chung LW (2008). Receptor activator of NF-κB Ligand (RANKL) expression is associated with epithelial to mesenchymal transition in human prostate cancer cells. Cell Research.

[bib33] Organ SL, Tsao MS (2011). An overview of the c-MET signaling pathway. Therapeutic Advances in Medical Oncology.

[bib34] Paland N, Kamer I, Kogan-Sakin I, Madar S, Goldfinger N, Rotter V (2009). Differential influence of normal and cancer-associated fibroblasts on the growth of human epithelial cells in an *in vitro* cocultivation model of prostate cancer. Molecular Cancer Research.

[bib35] Park SI, Soki FN, McCauley LK (2011). Roles of bone marrow cells in skeletal metastases: no longer bystanders. Cancer Microenvironment.

[bib36] Pathak S, Nemeth MA, Multani AS, Thalmann GN, von Eschenbach AC, Chung LW (1997). Can cancer cells transform normal host cells into malignant cells?. British Journal of Cancer.

[bib37] Portales-Casamar E, Kirov S, Lim J, Lithwick S, Swanson MI, Ticoll A, Snoddy J, Wasserman WW (2007). PAZAR: a framework for collection and dissemination of *cis*-regulatory sequence annotation. Genome Biology.

[bib38] Qi J, Nakayama K, Cardiff RD, Borowsky AD, Kaul K, Williams R, Krajewski S, Mercola D, Carpenter PM, Bowtell D (2010). Siah2-dependent concerted activity of HIF and FoxA2 regulates formation of neuroendocrine phenotype and neuroendocrine prostate tumors. Cancer Cell.

[bib39] Qian BZ, Li J, Zhang H, Kitamura T, Zhang J, Campion LR, Kaiser EA, Snyder LA, Pollard JW (2011). CCL2 recruits inflammatory monocytes to facilitate breast-tumour metastasis. Nature.

[bib40] Reimand J, Arak T, Vilo J (2011). g:Profiler – a web server for functional interpretation of gene lists (2011 update). Nucleic Acids Research.

[bib41] Sanbe T, Tomofuji T, Ekuni D, Azuma T, Irie K, Tamaki N, Yamamoto T, Morita M (2009). Vitamin C intake inhibits serum lipid peroxidation and osteoclast differentiation on alveolar bone in rats fed on a high-cholesterol diet. Archives of Oral Biology.

[bib42] Severin J, Waterhouse AM, Kawaji H, Lassmann T, van Nimwegen E, Balwierz PJ, de Hoon MJ, Hume DA, Carninci P, Hayashizaki Y (2009). FANTOM4 EdgeExpressDB: an integrated database of promoters, genes, microRNAs, expression dynamics and regulatory interactions. Genome Biology.

[bib43] Sheridan C, Kishimoto H, Fuchs RK, Mehrotra S, Bhat-Nakshatri P, Turner CH, Goulet R, Badve S, Nakshatri H (2006). CD44^+^/CD24^−^ breast cancer cells exhibit enhanced invasive properties: an early step necessary for metastasis. Breast Cancer Research.

[bib44] Smith DC, Smith MR, Sweeney C, Elfiky AA, Logothetis C, Corn PG, Vogelzang NJ, Small EJ, Harzstark AL, Gordon MS (2013). Cabozantinib in patients with advanced prostate cancer: results of a phase II randomized discontinuation trial. Journal of Clinical Oncology.

[bib45] Storey J, Tibshirani R, Brownstein MJ, Khodursky AB (2003). Statistical methods for detecting differential gene expression. Functional Genomics: Methods and Protocols.

[bib46] Subramanian A, Tamayo P, Mootha VK, Mukherjee S, Ebert BL, Gillette MA, Paulovich A, Pomeroy SL, Golub TR, Lander ES (2005). Gene set enrichment analysis: a knowledge-based approach for interpreting genome-wide expression profiles. PNAS.

[bib47] Tan W, Zhang W, Strasner A, Grivennikov S, Cheng JQ, Hoffman RM, Karin M (2011). Tumour-infiltrating regulatory T cells stimulate mammary cancer metastasis through RANKL–RANK signalling. Nature.

[bib48] Thomas GP, Baker SU, Eisman JA, Gardiner EM (2001). Changing *RANKL/OPG* mRNA expression in differentiating murine primary osteoblasts. Journal of Endocrinology.

[bib49] Verras M, Lee J, Xue H, Li TH, Wang Y, Sun Z (2007). The androgen receptor negatively regulates the expression of c-Met: implications for a novel mechanism of prostate cancer progression. Cancer Research.

[bib50] Wu Z, Irizarry R, Gentleman R, Murillo F, Spencer F (2004). A model based background adjustment for oligonucleotide expression arrays. Journal of the American Statistical Association.

[bib51] Xu J, Wang R, Xie ZH, Odero-Marah V, Pathak S, Multani A, Chung LW, Zhau HE (2006). Prostate cancer metastasis: role of the host microenvironment in promoting epithelial to mesenchymal transition and increased bone and adrenal gland metastasis. Prostate.

[bib52] Yang X, Shi C, Tong R, Qian W, Zhau HE, Wang R, Zhu G, Cheng J, Yang VW, Cheng T (2010). Near IR heptamethine cyanine dye-mediated cancer imaging. Clinical Cancer Research.

[bib53] Zhao F, Xuan Z, Liu L, Zhang MQ (2005). TRED: a Transcriptional Regulatory Element Database and a platform for *in silico* gene regulation studies. Nucleic Acids Research.

[bib54] Zhau HE, Odero-Marah V, Lue HW, Nomura T, Wang R, Chu G, Liu ZR, Zhou BP, Huang WC, Chung LW (2008). Epithelial to mesenchymal transition (EMT) in human prostate cancer: lessons learned from ARCaP model. Clinical & Experimental Metastasis.

